# Acceptability of mandatory vaccination against influenza, measles, pertussis and varicella by workers in healthcare facilities: a national cross-sectional study, France, 2019

**DOI:** 10.1186/s13690-023-01069-4

**Published:** 2023-04-05

**Authors:** Sophie Vaux, Laure Fonteneau, Muriel Péfau, Anne-Gaëlle Venier, Arnaud Gautier, Sophan Soing Altrach, Pierre Parneix, Daniel Levy-Bruhl

**Affiliations:** 1 Santé Publique France, Saint-Maurice, France; 2 Nouvelle Aquitaine Healthcare-Associated Infection Control Centre, Bordeaux University Hospital, Bordeaux, France

**Keywords:** Mandatory vaccination, Acceptability, Measles, Varicella, Pertussis, Influenza, Healthcare workers

## Abstract

**Background:**

Vaccination of healthcare workers (HCW) aims to protect them and to reduce transmission to susceptible patients. Influenza, measles, pertussis, and varicella vaccinations are recommended but not mandatory for HCW in France. Insufficient vaccine coverage for these diseases in HCW has raised the question of introducing mandatory vaccination. We conducted a survey to estimate acceptability of mandatory vaccination for these four vaccines by HCW working in healthcare facilities (HCF) in France, and to identify associated determinants.

**Methods:**

In 2019, we performed a cross-sectional survey of physicians, nurses, midwives and nursing assistants working in HCF in France using a randomised stratified three-stage sampling design (HCF type, ward category, HCW category). Data were collected in face-to-face interviews using a tablet computer. We investigated the possible determinants of acceptability of mandatory vaccination using univariate and multivariate Poisson regressions, and estimated prevalence ratios (PR).

**Results:**

A total of 8594 HCW in 167 HCF were included. For measles, pertussis, and varicella, self-reported acceptability of mandatory vaccination (very or quite favourable) was 73.1% [CI95%: 70.9–75.1], 72.1% [69.8–74.3], and 57.5% [54.5–57.7], respectively. Acceptability varied according to i) HCW and ward category for these three vaccinations, ii) age group for measles and pertussis, and iii) sex for varicella. For mandatory influenza vaccination, acceptability was lower (42.7% [40.6–44.9]), and varied greatly between HCW categories (from 77.2% for physicians to 32.0% for nursing assistants).

**Conclusion:**

HCW acceptability of mandatory vaccination was high for measles, pertussis and varicella but not as high for influenza. Vaccination for COVID-19 is mandatory for HCW in France. Replication of this study after the end of the COVID-19 crisis would help assess whether the pandemic had an impact on their acceptability of mandatory vaccination, in particular for influenza.

**Supplementary Information:**

The online version contains supplementary material available at 10.1186/s13690-023-01069-4.

## Background

Vaccine recommendations for healthcare workers (HCW) in France aim to protect them from communicable diseases, and to reduce transmission to susceptible patients. Some vaccinations are mandatory by law for HCW (diphtheria, tetanus, poliomyelitis, hepatitis B, COVID-19) while others are only recommended. The latter include influenza (recommended vaccination for HCW introduced in 2000), measles, pertussis (both in 2004), and varicella (2005). These four vaccinations are recommended for all HCW, and especially professionals providing care to most-at-risk patients.

In France, one dose of trivalent measles-mumps-rubella (MMR) vaccine is recommended for unvaccinated HCW born before 1980 with no history of measles, especially HCW working in wards with patients at risk of severe measles (i.e., immunosuppressed patients). A second dose is recommended for HCW born after 1980.

Vaccination against pertussis is recommended for HCW working in close and repeated contact with infants under six months of age (maternity, neonatology and paediatric services). Booster doses are recommended at 25, 45 and 65 years of age.

Vaccination against varicella is recommended for HCW with no history of varicella and a negative serology, especially those working in wards with patients at risk of severe varicella (i.e., immunosuppressed patients and patients in obstetrics and gynaecology, neonatology, paediatric, infectious diseases, and nephrology wards).

Finally, annual vaccination against seasonal influenza is recommended for all HCW [1].

Despite these recommendations, nosocomial outbreaks of these diseases still occur in France. One example is the two major measles epidemics in 2010–2011 and in 2018, both of which were associated with infections in heathcare facilities (HCF) in patients and HCW [2-4]. In 2009, vaccination coverage (VC) in HCW for these non-mandatory vaccinations was low: measles: 49.7% [30.8–68.8], pertussis: 11.4% [6.1–20.2], varicella: 29.9% [16.8–47.4] and influenza: 25.6% [14.7–40.6]. Instead, VC was high for mandatory vaccinations [5].

The low VC of non-mandatory vaccinations in HCW, and the involvement of these professionals in recent measles and influenza outbreaks led French health authorities to consider making vaccination mandatory for HCW for the four diseases listed above. In 2016, the Ministry of Health asked the French Immunization Technical Advisory Group for advice on this matter. The committee concluded that the measles, pertussis and varicella vaccines—but not the influenza vaccine—met the criteria for HCW mandatory vaccination [6]. In this context, exploring HCW acceptability of the extension of mandatory vaccination to these diseases would be useful. The present study, conducted in 2019, aimed to estimate this acceptability in HCW working in HCF in France. The secondary objective was to identify related determinants for each of the four diseases.

## Methods

### Participants and survey design

We performed a cross-sectional survey of physicians, nurses, midwives and nursing assistants in hospitals and private clinics throughout all French regions (i.e., 13 administrative regions in metropolitan France, and five overseas regions (Guadeloupe and Martinique in the Caribbean sea, French Guiana in South America, Reunion Island and Mayotte islands in the Indian Ocean). HCW employed for less than three months and student HCW (i.e., medical and nursing students) were not included.

The targeted HCF included all categories of hospitals (local, regional, medium-sized and university hospitals, clinics, cancer control centres, follow-up and rehabilitation facilities, and long-term care facilities). HCF with fewer than 30 beds, specific HCF such as haemodialysis or outpatient facilities, military hospitals, thermal imaging and radiological clinics, psychiatric hospitals and nursing homes (retirement homes) were all excluded.

HCW were selected using a randomised stratified three-stage sampling design. The first stage involved sampling of HCF from France’s national exhaustive administrative database (*La statistique annuelle des établissements de santé*, SAE December 2017, https://www.sae-diffusion.sante.gouv.fr) stratifying by type and size of facility (local hospitals, regional hospitals (< 300 beds, ≥ 300 beds), university hospitals, clinics (< 100 beds, ≥ 100 beds), cancer control centres, follow-up and rehabilitation facilities, and long-term care facilities), and by region, while taking the number of HCF in each region into account. In the second stage, wards were selected by sampling with stratification according to the following five categories: 1. ‘medicine or surgery wards for adults’, 2. ‘intensive care, oncology, haematology’, 3. ‘paediatric wards’, 4. ‘gynaecology and obstetrics wards’ and 5. ‘follow-up care and rehabilitation wards’. At least one ward in each category had to be included. Accordingly, at least five wards had to be included for each HCF but no more than 10. If a ward category was missing in a HCF, an extra ward was randomly drawn from another existing category. In the third stage, HCW were selected according to the working day in the HCF. More specifically, in a given ward, the survey was performed on a pre-defined day (weekends excluded). HCW working in the ward on that day were invited to participate.

### Organization

*Santé publique France*, the French national public health agency, coordinated the survey at the national level in collaboration with the care-associated infection prevention centre (CPias) [7] in the Nouvelle Aquitaine region.

In each HCF, one survey coordinator organized the survey. This person was either a member of the HCF hygiene team (local infection control practitioners) or an occupational health doctor. Interviewers performed face-to-face interviews with participating HCW using a standardized questionnaire. More information including questionnaires and user guide are available [8]. A tablet computer was provided to each survey coordinator in each HCF to conduct the interview.

In order to avoid desirability bias, respondents could choose to directly enter their answers themselves in the tablet computer, if they did not want the interviewer to know what their responses were.

### Sample size

We aimed to achieve a target sample size of 6200 HCW for metropolitan France and 7600 for France including overseas territories, based on a maximum acceptability rate for mandatory vaccination of 50%, a design effect of 2, an alpha error of 0.05 and a precision of at least 5% for the acceptability rate for HCW and for physicians in particular. The acceptability rate of 50% was chosen as it resulted in the largest sample size.

Assuming a HCF study participation rate of approximately 60%, an average of eight HCW per ward (i.e., 40 per HCF), and at least eight HCF included per region in metropolitan France, we randomized 274 HCF (229 HCF in metropolitan France, 45 in overseas territories) in order to obtain our desired sample size.

### Data collection and analysis

Individuals had to first provide their consent to participate by checking a consent box on the standardised web questionnaire before being able to open and complete the questionnaire. The following data were collected during interviews: demographic information (sex, age group: under 30 years, 30 to 39 years, 40 to 49 years, and 50 years or over; HCW category: physician, nurse, midwife and nursing assistant), administrative information (ward category and number of the ward (e.g., ranging from 1 to 5 when five wards were included); level of acceptability of mandatory vaccination for HCW for each of the four studied diseases (measles, varicella, pertussis and influenza). For the latter, the following question was used: “Are you in favour of mandatory vaccination for HCW’ for: 1.measles 2.pertussis 3. varicella 4. influenza? The possible answers for each disease were: 1.’very favourable’, 2. ‘quite favourable’, 3. ‘not very favourable’, 4. ‘not at all favourable’, 5. ‘I don’t know’. Questions were also asked about HCW vaccination status against measles (at least one dose), varicella, pertussis (at least one booster dose in adulthood) and influenza (2018–2019 season). For measles and varicella, vaccine coverage indicators were estimated for HCW who reported no history of the relevant disease and HCW who were unsure whether they had had it.

For HCW who refused to participate, few data were collected by the interviewers (sex, age group, HCW category, ward category, and ward number).

In order to estimate the sampling weights, we calculated the total number of wards by category in the HCF, and the total number of HCW working in these wards by HCW category (i.e., physicians, nurses, midwives and nursing assistants). The sampling design took into account all three sampling stages. Post-stratification adjustment for HCW category was applied taking into account region, sex and age.

To analyse acceptability of mandatory vaccination, a summary variable was constructed. Only measles, pertussis and varicella were concerned by this variable. Influenza vaccination was not included because of the disease’s annual characteristics and the different acceptability results for it. The summary variable had the following four modalities: i) ‘Quite or very favourable’ to mandatory vaccination for all three diseases; ii) ‘Not very or not at all favourable’ to mandatory vaccination for all three; iii) ‘Different answers’ if the respondent responded quite or very favourable for at least one vaccination but *either* not very or not at all favourable *or* ‘I don’t know’ for at least one other vaccination; iv) ‘I don’t know’ if the respondent answered ‘I don’t know’ for all three diseases.

The following possible determinants of acceptability of mandatory vaccination for HCW were explored through univariate and multivariate Poisson regressions: HCW category, age group, sex, ward category and region (18 administrative regions). All determinants with a *p*-value < 0.2 in the univariate analysis were introduced in the multivariate model. Final multivariate analysis models were built using backward elimination. Variables were considered significant at a *p*-value < 0.05, and only significant variables were kept in the final multivariate analyses. Prevalence ratios (PR), adjusted PR (PRa) and their 95% confidence intervals were used as measures of association. Significant interactions between HCW category and both age and sex were identified in the influenza vaccination multivariate analysis, leading to an analysis stratified by HCW category. Fisher’s exact test was used to compare participating and non-participating HCW.

For each disease, the acceptability rate of mandatory vaccination was estimated and compared between HCW who declared they were vaccinated or had previously had the disease, and those unvaccinated or with no disease history.

Data analyses were performed using Stata 15 SE.64 (StataCorp, USA). All estimates were made using the ‘svy’ command. Outcomes were given in percentages with their 95% confidence intervals (95% CI).

## Result

### Characteristics of the sample

Of the 274 HCF invited, 167 agreed to participate (participation rate (PR): 61%).

A total of 8594 HCW were included in the survey (metropolitan France: 7484, overseas territories: 1110). There were 1238 (14.4%) physicians, 405 (4.7%) midwives, 3674 (42.8%) nurses and 3277 (38.1%) nursing assistants (Table 1). The overall male/female ratio was 0.18.

**Table 1:** Healthcare workers’ acceptability of mandatory vaccination against measles and pertussis for HCW, France, 2019

	**Acceptability of mandatory vaccination** **% [CI95%]**				
	**N**	**Quite or very favourable**	**PR [CI95%]**	**PRa [CI95%]**	***P*-value**
**Measles**					
**All HCW**	**8594**	**73.1 [70.9–75.1]**			
**HCW category**					
Physician	1238	85.2 [81.8–88.0]	Ref	Ref	
Midwife	405	82.3 [78.8–85.4]	0.9 [0.9–1.0]	0.9 [0.8–0.9]	0.064
Nurse	3674	**73.9 [71.4–76.3]**	**0.9 [0.8–0.9]**	**0.9 [0.8–0.9]**	** < 0.001**
Nursing assistant	3277	**66.6 [63.3–69.6]**	**0.8 [0.7–0.8]**	**0.8 [0.7–0.8]**	** < 0.001**
**Age group (years)**					
Under 30	1884	75.9 [72.7–78.8]	Ref	Ref	
30 to 39	2606	77.1 [74.5–79.6]	1.1 [0.97–1.1]	1.0 [0.9–1.0]	0.7
40 to 49	1981	73.4 [69.6–76.8]	1.0 [0.9–1.0]	1.0 [0.9–1.0]	0.1
50 and over	1932	**69.0 [65.5–72.3]**	**0.9 [0.9–0.97]**	**0.9 [0.8–0.9]**	** < 0.001**
**Gender**					
Women	7220	72.9 [70.4–75.3]	Ref		
Men	1302	74.9 [71.7–77.9]	1.0 [0.97–1.1]		
**Ward category**					
Medicine or surgery for adults	3011	73.4 [69.8–76.7]	Ref	Ref	
Intensive care, oncology, haematology	1661	74.7 [70.6–78.3]	1.1 [0.9–1.1]	1.0 [0.9–1.1]	0.6
Gynaecology and obstetrics	1028	79.7 [74.7–83.9]	1.1 [1.0–1.7]	1.1 [1.0–1.2]	0.1
Paediatric	783	**81.7 [75.9–86.4]**	**1.1 [1.0–1.2]**	**1.1 [1.0–1.2]**	**0.003**
Follow-up care and rehabilitation	2111	66.2 [61.3–70.6]	0.9 [0.8–0.98]	0.89 [0.9–1.1]	0.1
**Pertussis**					
**All HCW**	**8594**	**72.1 [69.8–74.3]**			
**HCW category**					
Physician	1238	81.4 [77.9–84.5]	Ref	Ref	
Midwife	**405**	**79.0 [74.6–82.9]**	**0.97 [0.9–1.0]**	**0.9 [0.8–0.9]**	**0.001**
Nurse	**3674**	**73.1 [70.3–75.7]**	**0.9 [0.9–0.9]**	**0.9 [0.9–0.9]**	** < 0.001**
Nursing assistant	**3277**	**66.3 [62.9–69.6]**	**0.8 [0.8–0.9]**	**0.8 [0.8–0.9]**	** < 0.001**
**Age group (years)**					
Under 30	1884	75.7 [72.8–78.4]	Ref	Ref	
30 to 39	2606	76.1 [73.4–78.5]	1.0 [1.0–1.0]	1.0 [0.9–1.0]	0.5
40 to 49	**1981**	**71.3 [67.9–74.5]**	**0.9 [0.9–1.0]**	**0.9 [0.9–0.98]**	**0.006**
50 and over	**1932**	**68.4 [63.7–73.7]**	**0.9 [0.8–1.0]**	**0.9 [0.8–0.9]**	** < 0.001**
**Gender**					
Women	7220	71.9 [69.5–74.2]	Ref		
Men	1302	73.0 [69.6–76.1]	1.0 [1.0–1.1]		
**Ward category**					
Medicine or surgery for adults	3011	71.1 [67.6–74.4]	Ref	Ref	
Intensive care, oncology, haematology	1661	73.3 [68.1–78.0]	1.0 [1.0–1.1]	1.0 [0.96–1.1]	0.4
Gynaecology and obstetrics	**1028**	**80.5 [75.6–84.6]**	**1.1 [1.1–1.2]**	**1.1 [1.1–1.2]**	**0.001**
Paediatric	**783**	**83.2 [78.7–86.8]**	**1.0 [1.1–1.2]**	**1.2 [1.1–1.2]**	** < 0.001**
Follow-up care and rehabilitation	2111	66.8 [62.0–71.3]	0.7 [0.9–1.0]	1.0 [0.9–1.0]	0.4

Region was taken into account in multivariate analysis, with the Auvergne-Rhône-Alpes region taken as reference

Measles: differences were significant in the Ile-de-France region (PRa: 1.1 [1.0–1.3], *p* = 0.009, Mayotte region: 1.2[1.1–1.3], *p* < 0.001), Nouvelle-Aquitaine region: 1.2[1.1–1.4], *p* < 0.001), Réunion island region: 1.2 [1.1–1.3], *p* < 0.001)

Pertussis: differences were significant in the Ile-de-France region (PRa: 1.1 [1.0–1.3], *p* = 0.02), Martinique region: 1.2 [1.0–1.4], *p* = 0.01, Mayotte region: 1.3 [1.1–1.4], *p* < 0.001, Normandy region: 1.2 [1.0–1.4], *p* = 0.008, Nouvelle-Aquitaine region: 1.3 [1.1–1.4], *p* < 0.001, Réunion region: 1.1 [1.0–1.3], *p* = 0.008

Non-participation of HCW was lower than anticipated (234 HCW, 3%). The proportions of nurses, HCW under 30 years old, females, and those working in gynaecology and obstetrics wards were higher in participating than in non-participating HCW (Supplementary material, Table A).

A total of 721 wards were included. The total number of each ward category and the number of HCW working in each ward category were as follows: ‘medicine or surgery for adults’: 276 wards, 3011 HCW, ‘intensive care, oncology, haematology’: 120, 1661, ‘paediatric’: 66, 783,’gynaecology and obstetrics’: 72, 1028 and ‘follow-up care and rehabilitation’: 187, 2111.

### Mandatory vaccination against measles

When considering all HCW, 42.7% [40.2–45.2] of HCW were ‘very favourable’ to mandatory vaccination for HCW against measles, 30.4% [28.3–32.6] ‘quite favourable’, (or a total of 73.1% [70.9–75.1] for ‘quite or very favourable’), 6.6% [5.7–7.5] ‘not very favourable’, 9.4% [8.0–10.9] ‘not at all favourable’, and 11.0% [9.6–12.6] did not know.

Higher acceptability rates were observed in HCW who declared they were vaccinated against measles or had a history of measles compared to unvaccinated HCW and those with no measles history (75.9% [73.8–77.8] vs. 56.7 [50.6–62.5], PR: 1.3 [1.2–1.5], *p* < 0.001).

In the multivariate analysis, physicians were more likely to be ‘quite or very favourable’ to mandatory vaccination against measles than nurses and nursing assistants. HCW under 30 years old were more likely to be ‘quite or very favourable’ than those aged 50 and over. HCW working in paediatric wards were more likely to be ‘quite or very favourable’ than those working in medicine or surgery wards for adults (Table 1). Acceptability rates also differed between regions. An additional Figure file shows regional data (see Fig. 1a Additional file 1).

### Mandatory vaccination against pertussis

When considering all HCW, 41.0% [38.5–43.7] of those working in HCF were ‘very favourable’ to mandatory vaccination for HCW against pertussis, 31.0% [28.9–33.3]) ‘quite favourable’, (72.1% [69.8–74.3]) ‘quite or very favourable’), 6.3% [5.7–6.9] ‘not very favourable’, 9.0% [7.7–10.5] ‘not at all favourable’, and 12.7% [11.2–14.3] did not know.

Higher acceptability rates were observed for HCW who declared they were vaccinated against pertussis than those not vaccinated (80.0% [78.1–81.9] vs. 62.6 [59.2–65.8], PR: 1.3 [1.2–1.3], *p* < 0.001).

In the multivariate analysis, physicians were more likely to be ‘quite or very favourable’ to mandatory vaccination against pertussis than midwives, nurses and nursing assistants. HCW under 30 were more likely to be ‘quite or very favourable’ than those aged 40 to 49 and those aged 50 and over. HCW working in gynaecology and obstetrics wards and those in paediatric wards were more likely to be ‘quite or very favourable’ than those working in ‘medicine or surgery wards for adults’ (Table 1). Acceptability rates differed between regions (see Fig. 1b. Additional file 1).

### Mandatory vaccination against varicella

When considering all HCW, 28.4% [24.6–30.6] were ‘very favourable’ to mandatory vaccination against varicella, 29.0% [27.1–31.0]) ‘quite favourable’, (57.5% [54.5–57.7] quite or very rather favourable), 11.1% [10.1–12.1] ‘not very favourable’, 15.4% [13.7–17.2] ‘not at all favourable’, and 16.1% [14.1–18.2] did not know.

Higher acceptability rates were observed for HCW who declared they were vaccinated against varicella or those with a history of varicella (59.0% [56.0–61.8]) compared to unvaccinated HCW and those with no history (44.0% [37.1–51.2], PR: 1.3 [1.2–1.6], *p* < 0.001).

In the multivariate analysis, physicians were more likely to be ‘quite or very favourable’ to mandatory vaccination against varicella than nursing assistants. Males were more likely to be ‘quite or very favourable’ than females. HCW working in gynaecology and obstetrics wards and those in paediatric wards were more likely to be ‘quite or very favourable’ than those working in medicine or surgery wards for adults (Table 2). Acceptability rates differed between regions (see Fig. 1c Additional file 1).

**Table 2:** Healthcare workers’ acceptability of mandatory vaccination against varicella for HCW, France, 2019

Varicella	Acceptability of mandatory vaccination against varicella % [CI95%]				
	**N**	**Quite or very favourable**	**PR [CI95%]**	**PRa [CI95%]**	***P*-value**
**All HCW**	**8594**	**57.5 [54.5–57.7]**			
**HCW category**					
Physician	1238	65.2 [61.0–69.2]	Ref	Ref	
Midwife	405	67.9 [63.4–72.1]	1.0 [1.0–1.1]	1.0 [0.9–1.1]	0.9
Nurse	3674	57.5 [53.9–61.0]	0.9 [0.8–0.9]	0.9 [0.9–1.0]	0.15
Nursing assistant	3277	**54.1 [50.4–57.7]**	**0.8 [0.8–0.9]**	**0.9 [0.8–0.9]**	**0.01**
**Age group (years)**					
Under 30	1884	56.6 [52.5–60.6]	Ref		
30 to 39	2606	58.1 [55.6–61.1]	1.0 [1.0–1.1]		
40 to 49	1981	61.3 [57.8–64.7]	1.1 [1.0–1.2]		
50 and over	1932	56.3 [50.7–61.7]	1.0 [0.9–1.1]		
**Gender**					
Women	7220	56.2 [52.8–59.5]	Ref	Ref	
Men	1302	**65.4 [61.7–69.0]**	**1.2 [1.1–1.3]**	**1.2 [1.1–1.2]**	** < 0.001**
**HCF category**					
Hospital (< 300 beds)	3777	58.0 [53.1–62.6]	Ref		
University Hospital Centre	1640	60.3 [55.9–64.6]	1.0 [0.9–1.2]		
Cancer control Centre	835	58.9 [52.9–64.2]	1.0 [0.9–1.1]		
Medicine surgery obstetric centre	1572	51.9 [46.9–56.9]	0.9 [0.8–1.0]		
LTCF, Follow-up care and rehabilitation Centre	770	50.2 [39.4–56.9]	0.9 [0.7–1.1]		
**Ward category**					
Medicine or surgery for adults	3011	56.9 [52.5–61.1]	Ref	Ref	
Intensive care, oncology, haematology	1661	58.9 [52.7–64.8]	1.0 [0.9–1.2]	1.0 [0.9–1.1]	0.6
Gynaecology and obstetric	1028	**67.1 [61.9–72.0]**	**1.2 [1.1–1.3]**	**1.2 [11.0–1.3]**	**0.008**
Paediatric	783	**65.6 [58.0–72.5]**	**1.2 [1.0–1.3]**	**1.2 [1.1–1.3]**	**0.001**
Follow-up care and rehabilitation	2111	52.1 [47.3–56.8]	0.9 [0.8–1.0]	0.9 [0.8–1.0]	0.2

Region was taken into account in multivariate analysis with the Auvergne-Rhône-Alpes region taken as reference. Differences are significant in the Centre Val de Loire region (PRa: 1.3 [1.0–1.7], *p* = 0.04), Ile-de-France region (1.4 [1.1–1.8], *p* = 0.017), Martinique region (1.5 [1.1–1.9], *p* = 0.006), and the Nouvelle-Aquitaine region (1.4 [1.1–1.8], *p* = 0.02))

### Mandatory vaccination against measles and pertussis and varicella

When considering all HCW and the three diseases (measles and pertussis and varicella) for which vaccination acceptability was measured, 55.7% [52.6–58.7] were ‘quite or very favourable’ to mandatory vaccination for all three, 21.7% [19.1–24.6] had different answers depending on the disease, 13.4% [12.0–14.8] were ‘not very or not at all favourable’ for all three, while 9.3% [8.0–10.7] answered ‘I don’t know’ for all three (Table 3).

**Table 3:** Healthcare workers’ acceptability of mandatory vaccination against measles, pertussis and varicella for HCW, France, 2019

	**Acceptability of mandatory vaccination against measles and pertussis and varicella** **% [CI95%]**				
	**N**	**Quite or very favourable**	**Different answers**	**Not very or not at all favourable**	**I don’t know**
**All HCW**	**8594**	**55.7 [52.6–58.7]**	**21.7 [19.1–24.6]**	**13.4 [12.0–14.8]**	**9.3 [8.0–10.7]**
**HCW category**					
Physician	1238	62.9 [58.6–67.0]	25.4 [22.3–28.9]	6.2 [4.3–8.9]	5.5 [4.2–7.3]
Midwife	405	65.4 [60.5–70.0]	20.1 [16.2–24.5]	5.8 [4.5–7.5]	8.7 [6.5–11.7]
Nurse	3674	56.2 [52.5–59.8]	22.0 [19.0–25.4]	13.4 [11.6–15.3]	8.5 [7.0–10.2]
Nursing assistant	3277	51.5 [48.0–55.0]	19.9 [16.9–23.23]	16.3 [14.1–18.8]	12.3 [10.3–14.7]
**Age group (years)**					
Under 30	1884	54.2 [50.2–58.2]	25.6 [22.3–29.1]	12.2 [10.6–14.1]	8.0 [6.4–9.8]
30 to 39	2606	56.1 [53.0–59.0]	25.1 [22.5–27.8]	12.6 [10.9–14.5]	6.3 [5.2–7.6]
40 to 49	1981	59.1 [55.6–62.6]	18.5 [15.9–21.3]	12.7 [10.4–15.5]	9.7 [8.0–11.8]
50 and over	1932	55.4 [49.9–60.7]	18.6 [14.6–23.3]	15.0 [12.6–17.8]	11.1 [8.7–14.0]
**Gender**					
Women	7220	54.6 [51.2–58.0]	22.6 [19.7–26.0]	13.7 [12.2–15.4]	9.1 [7.7–10.7]
Men	1302	62.9 [58.3–67.3]	15.3 [11.6–19.9]	11.8 [9.9–13.9]	10.0 [8.2–12.2]
**Ward category**					
Medicine or surgery for adults	3011	55.1 [50.8–59.4]	22.3 [18.7–26.5]	13.7 [11.6–16.0]	8.9 [7.1–11.0]
Intensive care, oncology, haematology	1661	56.9 [50.9–62.7]	21.5 [17.7–25.7]	11.9 [8.6–16.3]	9.7 [7.8–12.1]
Gynaecology and obstetrics	1028	65.0 [59.2–70.4]	18.8 [15.0–23.3]	7.1 [4.8–10.4]	9.1 [7.1–11.4]
Paediatric	783	64.5 [56.9–71.4]	22.0 [16.7–28.5]	8.6 [5.4–13.4]	4.9 [2.6–8.7]
Follow-up care and rehabilitation	2111	50.2 [45.5–54.9]	21.1 [17.3–25.4]	17.1 [13.9–20.8]	11.6 [8.6–15.5]

### Mandatory vaccination against seasonal influenza

When considering all HCW, 19.9% [18.2–21.6] were ‘very favourable’ to mandatory vaccination for HCW against seasonal influenza, 22.9% [21.8–24.1] ‘quite favourable’ (42.7% [40.6–44.9] ‘quite or very favourable’), 16.2% [14.3–18.2] ‘not very favourable’, 27.9% [25.4–30.6] ‘not at all favourable’, and 13.2% [11.9–14.6] did not know.

Higher acceptability rates were observed for HCW who declared they were vaccinated against influenza during the 2018–2019 season compared to those unvaccinated (80.7% [78.1–83.1] vs. 22.4% [20.7–24.3], PR: 13.6 [3.3–3.9], *p* < 0.001).

In the multivariate analysis, physicians were more ‘quite or very favourable’ to mandatory vaccination against seasonal influenza than midwives, nurses and nursing assistants (Table 4). Differences were also observed between regions (see Fig. 1d Additional file 1).

**Table 4:** Healthcare workers’ acceptability of mandatory vaccination against influenza for HCW, France, 2019

	**Acceptability of mandatory vaccination for influenza** **(quite or very favourable)**																
	**All HCW**	**Physician**				**Midwife**				**Nurse**				**Nursing assistant**			
	**%** **[CI95%]**	**N**	**%** **[CI95%]**	**PRa** **[CI95%]**	***p***	**N**	**%** **[CI95%]**	**PRa** **[CI95%]**	***p***	**N**	**%** **[CI95%]**	**PRa** **[CI95%]**	***p***	**N**	**%** **[CI95%]**	**PRa** **[CI95%]**	***p***
All	42.7 [40.6–44.9]	1238	77.2 [71.2–82.2]	Ref		405	57.0 [52.3–61.6]	0.6 [0.5–0.7]	***	3674	42.0 [39.2–44.7]	0.3 [0.3–0.4]	***	3277	32.0 [29.5–34.5]	0.3 [0.2–0.3]	***
**Age group (years)**																	
< 30	33.3 [30.4–36.2]	128	96.4 [91.7–98.5]	Ref		83	57.5 [52.5–62.4]	Ref		1107	31.9 [28.1–35.9]	Ref		566	25.9 [21.2–31.3]	Ref	
30 to 39	43.6 [40.5–46.8]	386	83.5 [76.8–88.6]	0.9 [0.8–0.97]	*	131	53.5 [48.9–58.0]	0.9 [0.8–1.0]		1155	39.0 [35.3–42.9]	1.2 [1.1–1.4]	***	934	35.0 [29.8–40.4]	1.3 [1.1–1.6]	**
40 to 49 years	42.1 [39.0–45.3]	258	68.6 [58.2–77.4]	0.8 [0.6–0.9]	***	87	62.7 [54.6–70.2]	1.2 [1.0–1.3]	*	780	44.1 [39.6–48.6]	1.4 [1.2–1.6]	***	856	31.6 [27.6–36.0]	1.2 [1.0–1.5]	
50 and over	47.4 [42.9–52.0]	445	73.1 [61.5–82.1]	0.8 [0.7–0.9]	***	89	56.9 [52.8–60.9]	1.0 [0.8–1.3]		570	47.4 [41.2–53.8]	1.5 [1.3–1.9]	***	828	33.3 [28.4–38.5]	1.3 [1.0–1.6]	
**Gender**																	
Women	40.8 [38.5–43.2]	615	81.3 [71.9–85.7]			393	56.5 [52.0–60.9]	Ref		3267	40.8 [37.6–43.9]	Ref		2945	31.9 [29.0–35.0]		
Men	54.4 [50.4–58.4]	619	72.4 [63.1–80.1]			11	91.0 [88.9–92.6]	1.6 [1.5–1.7]	***	378	50.4 [44.1–56.7]	1.2 [1.1–1.4]	**	294	34.5 [25.9–44.2]		
**Ward category**																	
Medicine or surgery for adults	42.6 [39.7–45.6]	454	75.2 [67.1–81.9]	Ref						1484	40.4 [36.3–43.9]	Ref		1072	35.1 [31.3–39.2]	Ref	
Intensive care, oncology, haematology	49.2 [44.6–53.8]	264	79.9 [66.9–88.7]	1.0 [0.9–1.2]						890	51.9 [46.7–57.1]	1.3 [1.1–1.5]	***	506	31.6 [26.5–37.3]	0.9 [0.7–1.1]	
Gynaecology and obstetrics	50.9 [45.7–56.2]	127	84.5 [70.3–88.7]	1.1 [0.9–1.4]		405	57.0 [52.5–61.3]			81	52.6 [39.1–65.7]	1.1 [0.9–1.4]		419	32.2 [26.9–38.1]	0.8 [0.7–1.0]	
Paediatric	44.1 [37.1–51.3]	163	84.5 [70.3–92.6]	1.1 [1.0–1.2]	*					392	43.5 [34.8–52.5]	1.1 [0.9–1.4]		227	23.9 [18.2–30.6]	0.6 [0.5–0.8]	**
Follow-up care and rehabilitation	36.2 [32.3–40.4]		73.5 [71.3–82.1]	1.0 [0.9–1.2]						827	37.4 [31.2–43.9]	0.9 [0.8–1.1]		1053	28.5 [24.8–32.6]	0.8 [0.7–0.97]	*
**HCF size**																	
< 100 beds	44.1 [36.7–51.8]	196	82.7 [63.4–92.9]			36	38.1 [36.5–39.8]	Ref		787	42.1 [33.8–50.9]			603	34.1 [26.7–42.4]		
100 to 299 beds	38.9 [32.8–43.3]	335	64.7 [53.7–74.4]			88	35.4 [28.7–42.7]	0.8 [0.6–1.0]		1145	38.4 [31.1–46.1]			1056	29.4 [24.8–34.5]		
300 to 450 beds	43.3[38.3–48.5]	270	68.2 [51.6–81.2]			76	72.3 [65.8–77.9]	1.5 [0.9–2.6]		567	42.5 [34.9–50.4]			485	33.3 [27.6–39.6]		
> 450 beds	45.0 [41.6–48.4]	437	86.3 [80.3–90.7]			205	62.5 [54.6–69.7]	1.5 [1.0–2.3]	*	1175	43.7 [40.1–47.2]			1133	32.7 [28.1–37.7]		
**HCF category**																	
Hospital (< 300 beds)	42.7 [39.6–45.9]	603	73.1 [63.3–81.0]	Ref		218	64.4 [58.6–69.8]	Ref		1418	42.5 [38.3–46.9]			1538	32.4 [29.1–35.9]		
University Hospital Centre	45.7 [42.7–48.8]	250	85.3 [77.9–90.5]	1.1 [1.0–1.3]		81	44.4 [33.1–56.4]	0.6 [0.4–0.7]	***	720	45.1 [41.7–48.6]			589	31.8 [28.4–35.5]		
Cancer control Centre	43.6 [36.2–51.4]	140	91.9 [76.6–97.5]	1.3 [1.0–1.5]	*	0	-			452	37.3 [27.9–47.9]			243	28.5 [16.2–45.1]		
Medicine surgery obstetric centre	39.0 [32.2–46.3]	169	76.6 [61.5–87.1]	1.0 [0.8–1.3]		104	36.3 [33.4–39.2]	0.9 [0.6–1.4]		761	36.9 [29.5–45.0]			538	28.8 [21.836.8]		
LTCF, Follow-up care and rehabilitation Centre	36.8 [27.0–47.8]	76	78.7 [65.1–88.0]	1.1 [0.9–1.3]		2	-			323	34.2 [21.5–49.6]			369	32.8 [24.6–42.3]		

*p*-value: *: < 0.05; **: < 0.01; *** < 0.001; Region was taken into account in multivariate analysis with the Auvergne-Rhône-Alpes region taken as reference. Differences are significant: for physicians: in Reunion Island (PRa: 10.7 [0.5–0.9], *p* = 0.01); for midwives: Guiana: 1.7 [1.2–2.3], *p* < 0.001, Pays-de-la-Loire: 0.7: 0.6–0.96, *p* < 0.05) (Martinique and Mayotte: non available); for nurses: Bourgogne-Franche-Comté: 1.5 [1.2–2.0], *p* = 0.003, Ile-de-France: 1.5 [1.2–2.0], *p* = 0.001, Martinique: 1.4 [1.1–1.9], *p* = 0.004, Normandie: 1.3 [1.0–1.7], *p* < 0.05; nursing assistant: Guadeloupe: 1.3 [1.0–1.7], 0.04, Occitanie: 0.6 [0.4–0.9], *p* = 0.02, Pays-de-la-Loire: 0.7 [0.4–0.9], *p* = 0.02)

The level of acceptability in physicians under 30 years was very high (‘quite or very favourable’: 96.4% [91.7–98.5]). In the multivariate analysis, this age group was more likely to be ‘quite or very favourable’ than older physicians. Furthermore, physicians working in paediatric wards were more likely to be ‘quite or very favourable’ than those working in medicine or surgery wards for adults.

Among midwifes and nurses, males were more likely to be ‘quite or very favourable’ than females. However, the number of male midwives included was very small (*n* = 11).

Among nurses, acceptability of mandatory vaccination for influenza increased with age (PRa increase with age). Specifically, it was higher in those over 30 years old than in younger nurses. Acceptability was also higher in nurses working in intensive care, oncology, and haematology wards than in those working in medicine or surgery wards for adults.

With regard to nursing assistants, acceptability of mandatory vaccination was low especially in those under 30 years old and nursing assistants working in paediatric wards than in those working in medicine or surgery wards for adults (Table 4).

## Discussion

In France vaccinations against measles, pertussis, varicella and influenza are recommended but not mandatory for HCW. We estimated HCW acceptability of hypothetical mandatory vaccination for these four diseases in 2019.

Only a few studies to date have assessed HCW acceptability of mandatory vaccination and most focused exclusively on influenza vaccination [9]. HCW acceptability of mandatory vaccination in previous studies was usually high: in 2010–2011, 63% and 65% of HCW working, respectively, in primary healthcare centres and tertiary-care hospitals in Greece supported mandatory vaccination for all HCW. These figures were 80% and 100%, respectively, for interviewed HCW caring for immunocompromised patients [10, 11], nearly 70% for HCW working with vulnerable patients in England in 2013 [12], 91% in 2009 in HCW working in two tertiary-referral teaching hospitals in Australia [13], and 97% in HCW students in Athens [14].

### Measles, pertussis and varicella

With regard to measles, pertussis, and varicella, acceptability of mandatory vaccination differed: it was high for the first two at 73.1% [70.9–75.1] and 72.1% [69.8–74.3], respectively, but moderate for the latter (57.5% [54.5–57.5]).

Our HCW acceptability rates were higher than those published in other countries for measles, pertussis and varicella. More specifically, in Greece, 17% of HCW working in primacy healthcare centres in 2010 and 15% of HCW working in tertiary hospitals in 2012 favoured mandatory vaccination against measles for all HCW (for interviewed HCW caring for immunocompromised patients these values were 43% and 33%). For pertussis vaccination, these values were 13% and 11%, respectively (32% and 21%, respectively, for interviewed HCW caring for immunocompromised patients), and 12% and 17%, respectively, for varicella (32% and 32% for those caring for immunocompromised patients) [10, 11]. In Italy, HCW acceptability rates were 38.4% for measles, 39.8% for pertussis and 33.8% for varicella [15].

There are several likely reasons for high acceptability rates of mandatory vaccination for measles and pertussis in France. France experienced a dramatic measles outbreak between 2008 and 2011 with more than 22 000 reported cases, and 85 nosocomial outbreaks. HCW were involved in 85% of these episodes [3]. France experienced another measles outbreak in 2017 and a nationwide resurgence of pertussis in 2012–2013 [4, 16]. Training and communication were strengthened with the objectives of increasing vaccination coverage in HCW. In January 2018, the French government extended mandatory vaccinations for children from three (diphtheria, tetanus, poliomyelitis) to 11 diseases (pertussis, Haemophilus influenza b, hepatitis B, invasive pneumococcal diseases, meningococcal C, measles, mumps and rubella). Various actions were implemented during this period, including a commitment by the French government to encourage vaccination, citizen consultation on vaccination, and social debate. In this context, the fact that measles and pertussis vaccines are only recommended (i.e., not mandatory) for HCW—despite their being involved during nosocomial outbreaks—was an issue for debate. The combinaison of these events probably led to high rates of acceptability of mandatory vaccinations against measles and pertussis by HCW.

In our study, we showed that the acceptability rate for mandatory vaccination for each of these three diseases was higher in HCW who declared to be fully vaccinated. Multivariate analysis highlighted that acceptability rates for mandatory vaccination (‘quite or very favourable’) for the three diseases differed significantly according to HCW category. More specifically, they were significantly higher in physicians than in nursing assistants for all three [17].

Acceptability rates differed significantly according to age, with HCW older than 50 being less favourable to mandatory vaccination for measles and pertussis than those under 30. Determinants of vaccination coverage for measles, pertussis and varicella among HCW have been previously studied. Vaccination coverage against the three diseases were also higher in physicians than in nursing assistants and age and sex have been found to be determinants of vaccination coverage, particularly for pertussis [17]. Vaccination coverage and acceptability for mandatory vaccination shared broadly the same determinants. HCWs who accept to be vaccinated are most often those who support mandatory vaccination for HCW.

Rates also differed between ward categories. More specifically, acceptability was higher in wards with at-risk patients, and wards where HCW should be vaccinated as a priority [1]. For instance, for measles, it was higher in HCW working in paediatric wards, and for pertussis, it was higher in HCW working in paediatric, gynaecology and obstetrics wards. These results suggest a greater awareness of risks attached to nosocomial transmission originating from an unvaccinated health care worker that translated into higher vaccination coverage amongst professionals working in these wards. Higher acceptability for mandatory vaccination for pertussis among young HCW and those working with newborns and infants may also be partly related to the communication efforts promoting the cocooning strategy around newborns.

The majority of HCW (55.7%) were ‘quite or very favourable’ of mandatory vaccination for measles, pertussis and varicella. Only 13.4% of participants responded ‘not very favourable’ or ‘not at all favourable’ for all three vaccinations. Just over six percent (6.2%) of physicians systematically responded in this way, which was close to the percentage found in a 2005–2006 French study of general practitioners (8.1%) declaring their opposition to mandatory vaccination. However it was much lower than the 20.7% of paediatricians who responded in this way in that same study [18].

Almost 10 percent of HCW in our study responded “I don’t know” when asked about their acceptability of mandatory vaccination for these three diseases. Nursing assistants, females, HCW working in medicine or surgery wards for adults, and HCW working in follow-up care and rehabilitation wards were all less likely to be ‘quite of very favourable’ to mandatory vaccination (51.5%, 54.6%, 55.1%, and 50.2%, respectively). In metropolitan France, we also observed regional differences: acceptability rates globally followed a North-West (highest) South-East (for the lowest) divide. A similar divide was observed in a study measuring HCW vaccination coverage against measles, pertussis varicella, and influenza [8, 17], and is consistent with findings on acceptability of vaccination in the general public in France [19].

HCW vaccination for these three diseases is mandatory in very few countries. Measles vaccination is mandatory for HCW in Albania, Croatia, Portugal, and Slovenia. In Serbia and Finland, it is mandatory for HCW who care for at risk-patients [20, 21]. Pertussis vaccination is mandatory for HCW in Albania, Croatia, Portugal (for pregnant HCW only), and Slovenia (for HCW at greater risk fo exposure to pertussis). In Finland, vaccination against varicella is required for HCW working with at-risk patients [20].

### Influenza

In our study, overall HCW acceptability of mandatory vaccination for influenza was 42.7% [40.6–44.9] and was higher in HCW vaccinated against this disease [17]. In contrast, this overall rate was much lower than those observed for the other three diseases studied (see above). Our overall rate for influenza was also lower than values reported in Greece among HCW working in tertiary health hospitals (51% for all interviewed HCW, and 67% for interviewed HCW caring for immunocompromised patients [10]) and values from Australia (46.8%) [13]. A recent meta-analysis including 40 articles around the world, concluded that the proportion of HCW supporting mandatory vaccination against influenza was 61% [53–68%], with large variations observed between continents (from 54% in Europe to 69% in Asia). Based on these findings, acceptability in our study of French HCW was lower than the average European estimate. Moreover, only the United Kingdom (32.2%, 2011) [22], Italy (40.3%, 2017) [15], Belgium (33.0% for interviewed HCW working in hospitals, 41.9% for those working in nursing homes, 2018) [23], and Ireland (35.7%, 2018) [24] had lower influenza scores than ours. However, comparisons are difficult to make, because—as highlighted in our results and as reported in other studies [25]—acceptability rates vary greatly according to HCW category profession. In our study, acceptability of mandatory influenza vaccination varied from 77% for physicians to 32% for nursing assistants. Furthermore, HCW age was also a determinant of acceptability in our study, and was associated with HCW category. For example, acceptability increased with age in nurses, while for midwifes, age was not a determinant. For nursing assistants, acceptability tended to increase with age but was only significantly greater in 30–39 year-olds (vs. those under 30 years old). Finally, a very high acceptability rate (96.4%) was observed for physicians under 30. Similarly, the influence of sex and ward category varied according to HCW category.

Annual influenza vaccination is recommended for HCW in 30 European countries (either for all HCW or HCW in direct contact with patients or immunocompromised patients), in the USA and in Japan. In Serbia and in Finland, it is mandatory only for subgroups such as HCW working in HCF caring for high-risk patients [20, 21, 26]. However, no country has yet made it mandatory for all HCW. In the United States, mandatory influenza vaccination for HCW is supported by many scientific societies [27] and is associated with a vaccine coverage of almost 100% in structures where it is implemented [26, 28].

### Ethical considerations of mandatory vaccination for HCW

Previous studies showed that implementing mandatory vaccination increases vaccination coverage in the general population [29] and in HCW [26, 28]. However, the ethics of mandatory vaccination for HCW merit discussion. Any such debate must take into account the disease, the type of patients exposed, HCW categories, and the effectiveness and safety profile of the vaccine. Mandatory vaccination for HCW can only be justified for diseases that cause significant morbidity or mortality in the context of healthcare-associated infection transmission and if a safe and effective vaccine is available. Practical arguments for and against mandatory vaccination have been listed by various authors taking into account effectiveness, necessity, a false sense of security, administrative issues, cost, coercion, civil liberties, and potential harms [30, 31].

Previous studies, notably in New South Wales in Australia, showed that the implementation of mandatory vaccination should involve communication, leadership support, free of charge vaccination, easy access, and an appropriate data collection and reporting system [32].

The very high HCW acceptability rates for measles and pertussis vaccination in our study would suggest the usefulness of mandatory vaccination for these diseases, especially HCW working with at-risk patients in paediatric wards and those in gynaecology and obstetrics wards. Acceptability of mandatory varicella vaccination was lower in our study than for measles and pertussis, almost certainly because a very high proportion of respondents had a history of this disease. However, the rate we found may be underestimated if participating HCW did not take into account the fact that mandatory vaccination would only occur in the absence of a history of varicella.

With regard to influenza, voluntary influenza vaccination programmes for HCW in France have failed to lead to high uptake for decades [17, 33, 34]. Mandatory vaccination for HCW could be justified as it would reduce transmission and avoid disruption to healthcare services. However, the suboptimal effectiveness of influenza vaccines and the need for annual administration raise doubts about the value of such a policy. Furthermore, in a context where vaccination became mandatory, HCW who refused annual influenza vaccination would have to be assigned to other activities, which in turn would create difficulties in terms of staff management during the winter season. These different points could partly explain the lower acceptability of mandatory influenza vaccination which we observed in comparison to the other three diseases studied.

The regulations on mandatory vaccination of HCW have not changed after the 2016 French Immunization Technical Advisory Group (CTV) statement [6]. In late 2022, the French Ministry of Health has asked two committees to work on the list of vaccines that should remain or be made mandatory for HCWs (namely the National Advisory Ethics Committee for Health (CCNE) and the CTV). Our study results have been shared with them.

### Strengths and limitations

One of the main strengths of this study was the implementation of a random selection which took into account HCW category, ward category, and hospitals type, and the consideration of these sampling weights in all calculations. As a consequence, the estimated acceptability rates were independent of HCW category distribution, ward category distribution and HCF category distribution in the sample.

Randomly selection of HCW to participate in a study may be a complex procedure. The protocol for randomly selecting HCW should therefore be simple. In the present study, we assumed that randomly selecting one day in the working week, and interviewing all the HCW present in a specific ward in a HCF on that day, was an effective approach to ensure randomization. Accordingly, we assumed that HCW acceptability of mandatory vaccination was independent of the working day in the week.

Our study has limitations. First, acceptability of mandatory vaccination may have been influenced by desirability bias. However, to avoid this difficulty, a tablet computer was provided in each HCF to allow HCW to directly input answers if he/she did not want the interviewer to know what his/her answers were. Second, HCW less adherent to mandatory vaccination may have been more likely to refuse participation, leading to an overestimation of acceptability. However, the impact of this limitation would have small given the low percentage (3%) of HCW who refused to participate. Having said that, 3% seems excessively small. It is possible that interviewers did not systematically record refusals to participate. Unfortunately, the anonymous nature of the study prevents us from calculating the degree of this underestimation. Finally, the self-reported vaccination status may have introduced recall biais for vaccination status.

## Conclusion

The high percentages of HCW favourable to mandatory vaccination for HCW against measles (73.1%) and pertussis (72.1%) and the moderate percentage for varicella (57.5%) were the main findings of our study. More than half (55.7%) of the participating HCW were favourable to mandatory vaccination against all three of these diseases. HCW acceptability varied by HCW category; nevertheless, even in nursing assistants—the category least favourable—the acceptability rate was over 50% for all three diseases. HCW age was associated with acceptability of mandatory vaccination for measles and pertussis. For influenza vaccination, opinion differed greatly depending on HCW category (from 77.2% in physicians to 32.0% in nursing assistants; overall HCW: 42.7%). Higher acceptability rates for each disease were observed for HCW who declared they were vaccinated for the relevant disease.

This study was conducted before the COVID-19 health crisis, and the implementation of mandatory vaccination for this disease for all HCW in France. Conducting a similar study when the COVID-19 crisis ends could assess whether the pandemic has had an impact on HCW acceptability of mandatory vaccination for different diseases, in particular influenza.

## Supplementary Information

**Additional file 1: Table A.** Characteristics of participants and non-participants in the survey, France 2019. **Figure 1.** Healthcare workers’ (HCW) acceptability of mandatory vaccination (quite or very favourable) against measles, pertussis, varicella and influenza for HCW according to region; study in healthcare facilities, France, 2019.

## Data Availability

The datasets used and/or analyzed during the current study are available from the corresponding author on reasonable request.

## Abbreviations

HCW: Healthcare worker
HCF: Heathcare facilities
MMR: Measles-mumps-rubella
PR: Prevalence ratios
PRa: Adjusted prevalence ratios
VC: Vaccination coverage
