# Supplementary material for: Acceptability of mandatory vaccination against influenza, measles, pertussis and varicella by workers in healthcare facilities: a national cross-sectional study, France, 2019
Source: Arch Public Health. 2023 Apr 5;81:51. doi: 10.1186/s13690-023-01069-4 (PMC10076374; doi:10.1186/s13690-023-01069-4)
Supplement: Supplementary file 1 — Additional file 1: Table A. Characteristics of participants and non-participants in the survey, France 2019. Figure 1. Healthcare workers’ (HCW) acceptability of mandatory vaccination (quite or very favourable) against measles, pertussis, varicella and influenza for HCW according to region; study in healthcare facilities, France, 2019. [file 13690_2023_1069_MOESM1_ESM.docx]

Additional File_Supplementary Material

**Table A. Characteristics of participants and non-participants in the survey, France 2019**

|  |  | **Participants** | | | **Non-participants** | | |  |
| --- | --- | --- | --- | --- | --- | --- | --- | --- |
|  | **N** | | **Proportion (%)** | **Proportion among participants (%)** | **N** | **Proportion (%)** | **Proportion among non-participants (%)** | **P ^3)^** |
| **All HCW** | **8594** | | **97.3** | **100** | **234** | **2.7** | **100** |  |
| **HCW category** |  | |  |  |  |  |  |  |
| Physician | 1238 | | 96.7 | 14.4 | 42 | 3.3 | 17.9 | 0.1 |
| Midwife | 405 | | 98.8 | 4.7 | 5 | 1.2 | 2.1 | 0.07 |
| Nurse | 3674 | | 97.8 | 42.8 | 83 | 2.2 | 35.5 | 0.03 |
| Nursing assistant | 3277 | | 96.9 | 38.1 | 104 | 3.1 | 44.4 | 0.05 |
| **Age group (years) ^1)^** |  | |  |  |  |  |  |  |
| Under 30 | 1884 | | 98.8 | 21.9 | 22 | 1.2 | 9.4 | <0.001 |
| 30 to 39 | 2606 | | 97.3 | 30.3 | 72 | 2.7 | 30.8 | 0.9 |
| 40 to 49 | 1981 | | 97.0 | 23.1 | 61 | 3.0 | 26.1 | 0.3 |
| 50 and over | 1932 | | 97.1 | 22.5 | 58 | 2.9 | 24.8 | 0.4 |
| **Sex ^2)^** |  | |  |  |  |  |  |  |
| Female | 7220 | | 97.7 | 84.0 | 171 | 2.3 | 73.0 | <0.001 |
| Male | 1302 | | 96.1 | 15.2 | 53 | 3.9 | 22.7 | 0.003 |
| **Ward Category** |  | |  |  |  |  |  |  |
| Medicine or surgery for adults | 3011 | | 97.3 | 35.0 | 84 | 2.7 | 35.9 | 0.8 |
| Intensive care, oncology, haematology | 1661 | | 96.9 | 19.3 | 53 | 3.1 | 22.7 | 0.2 |
| Gynaecology and obstetrics | 1028 | | 98.8 | 12.0 | 13 | 1.2 | 5.6 | 0.002 |
| Paediatric | 783 | | 97.1 | 9.1 | 23 | 2.9 | 9.8 | 0.7 |
| Follow-up care and rehabilitation | 2111 | | 97.2 | 24.6 | 61 | 2.8 | 26.1 | 0.6 |

1. Age group unknown for 191 participating HCW (2.2%) and 21 non-participating HCW (9.0%). Gender unknown for 72 participating HCW (0.8%), and 10 non-participating HCW (4.3%).
2. Fisher’s exact test

Figure 1 [Supplementary Material]. Healthcare workers’ (HCW) acceptability of **mandatory vaccination (quite or very favourable) against measles, pertussis, varicella and influenza for HCW according to region; study in healthcare facilities, France, 2019**

| 1.a Vaccination against measles | | 1.b Vaccination against pertussis | |
| --- | --- | --- | --- |
| 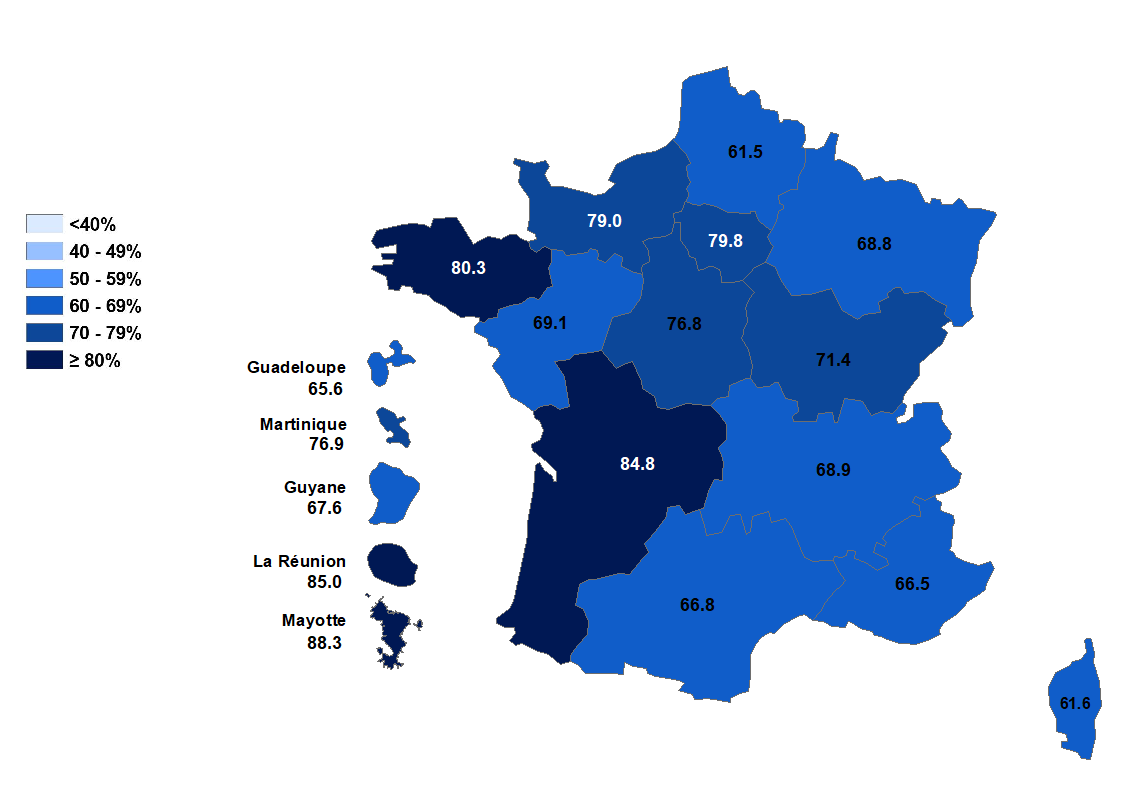 | 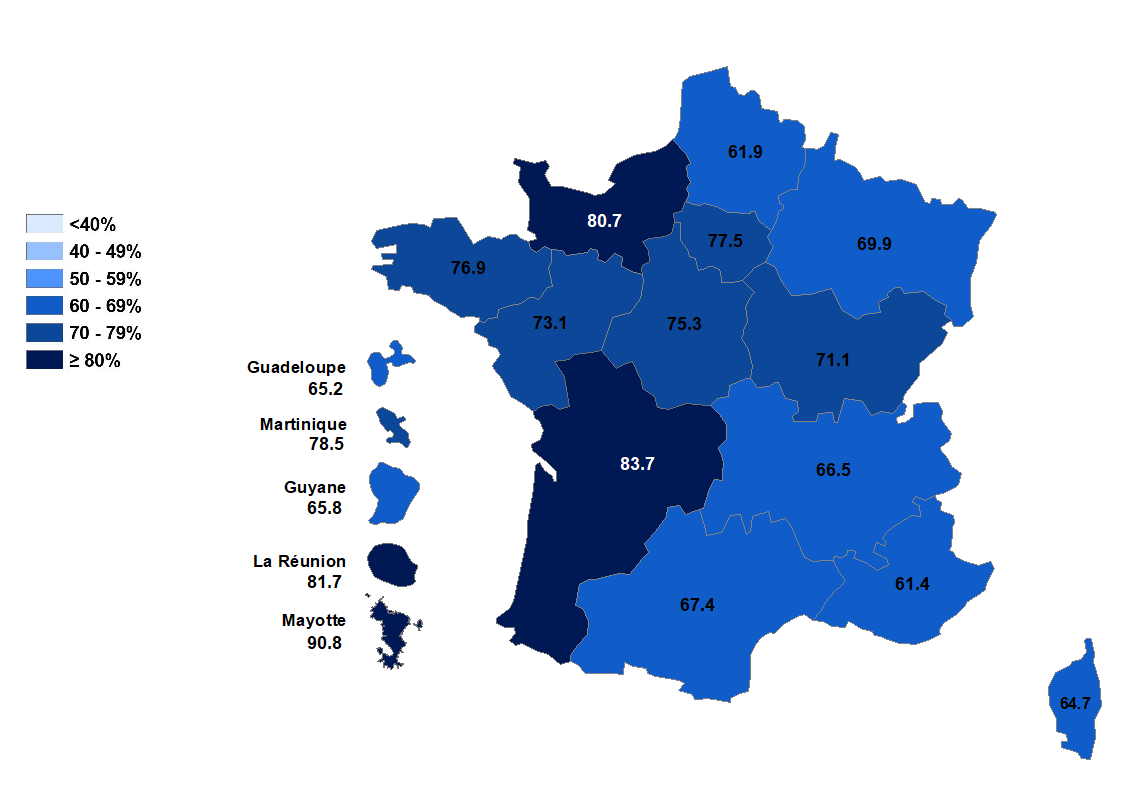 | |  |
| 1.c Vaccination against varicella | | 1.d Vaccination against influenza | |
| 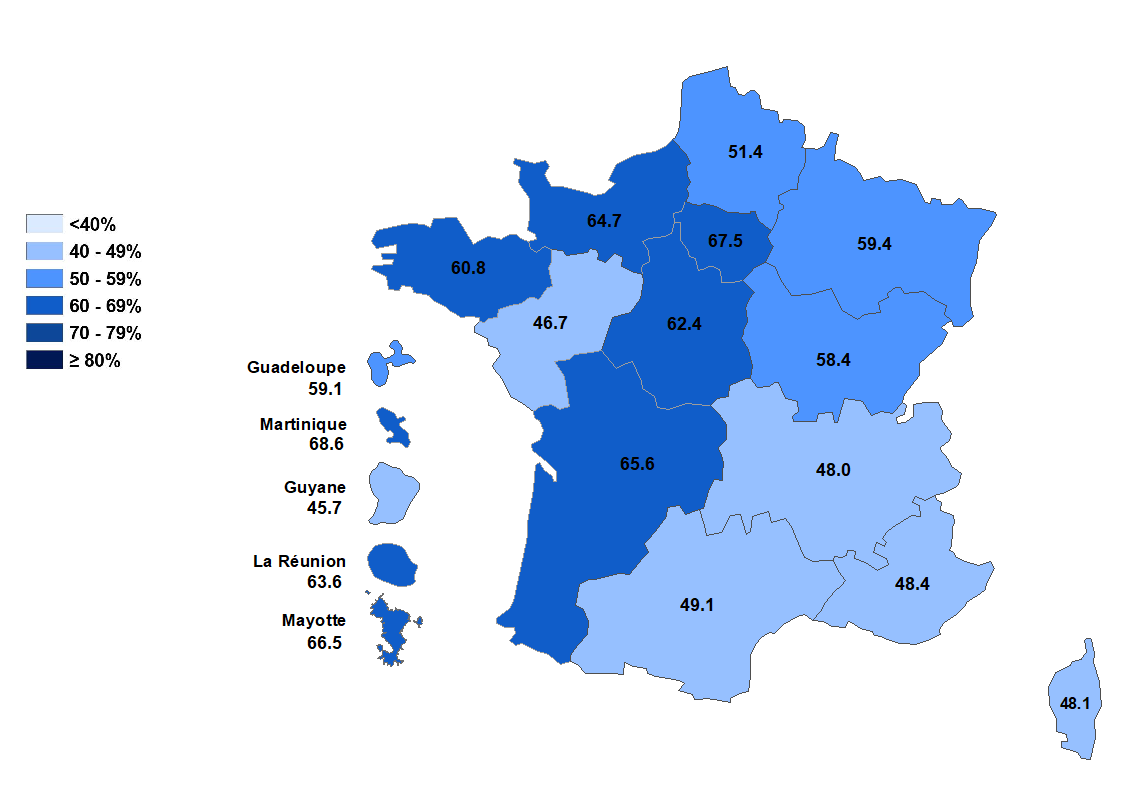 | | 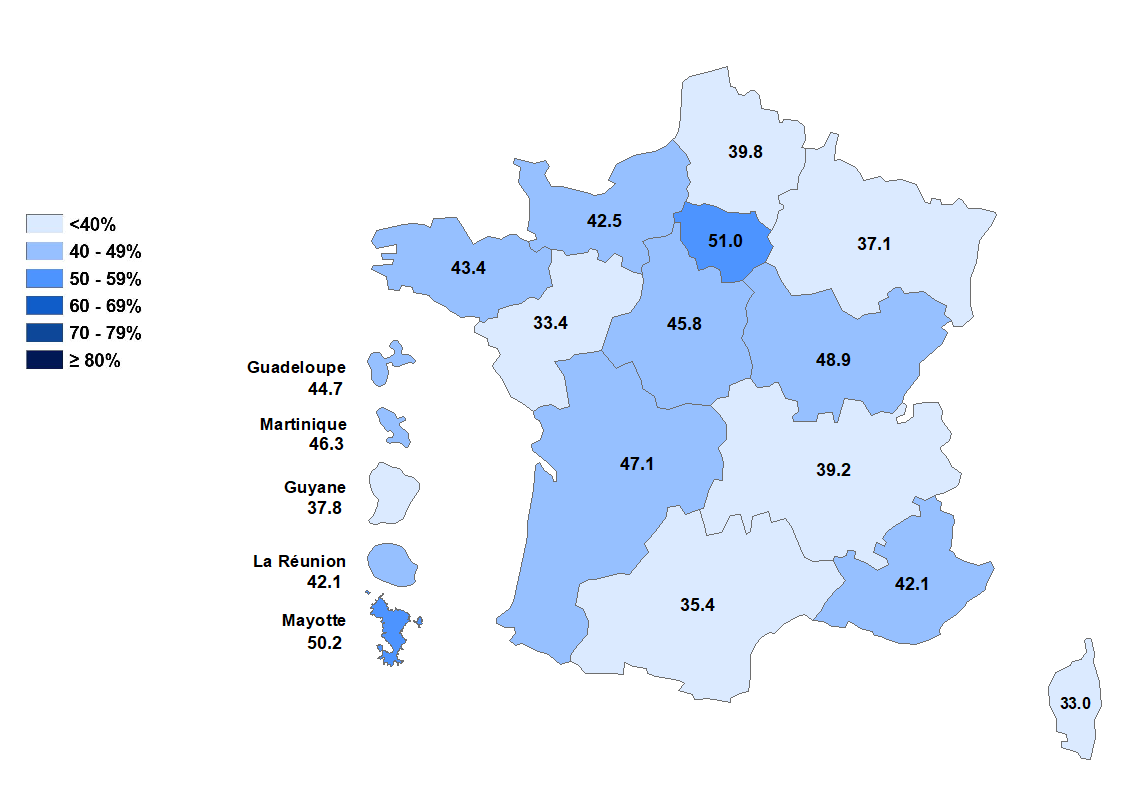 | |
